# Changes in Central Asia’s Water Tower: Past, Present and Future

**DOI:** 10.1038/srep35458

**Published:** 2016-10-20

**Authors:** Yaning Chen, Weihong Li, Haijun Deng, Gonghuan Fang, Zhi Li

**Affiliations:** 1 State Key Laboratory of Desert and Oasis Ecology, Xinjiang Institute of Ecology and Geography, Chinese Academy of Sciences, Urumqi 830011, China; 2 University of Chinese Academy of Sciences, Beijing 100049, China

## Abstract

The Tienshan Mountains, with its status as “water tower”, is the main water source and ecological barrier in Central Asia. The rapid warming affected precipitation amounts and fraction as well as the original glacier/snowmelt water processes, thereby affecting the runoff and water storage. The ratio of snowfall to precipitation (S/P) experienced a downward trend, along with a shift from snow to rain. Spatially, the snow cover area in Middle Tienshan Mountains decreased significantly, while that in West Tienshan Mountains increased slightly. Approximately 97.52% of glaciers in the Tienshan Mountains showed a retreating trend, which was especially obvious in the North and East Tienshan Mountains. River runoff responds in a complex way to changes in climate and cryosphere. It appears that catchments with a higher fraction of glacierized area showed mainly increasing runoff trends, while river basins with less or no glacierization exhibited large variations in the observed runoff changes. The total water storage in the Tienshan Mountains also experienced a significant decreasing trend in Middle and East Tienshan Mountains, but a slight decreasing trend in West Tienshan Mountains, totally at an average rate of −3.72 mm/a. In future, water storage levels are expected to show deficits for the next half-century.

In arid and semi-arid regions, mountains are important water suppliers, as they feed most of the local rivers^1^,^2^,^3^. There is generally a relative abundance of precipitation in mountains, stored in the form of glaciers, snow and permafrost^4^,^5^,^6^. A mountain’s role as water tower is especially crucial in arid regions. Snow and glacial melt are important hydrologic processes in these areas, as supplies of solid water are a key element in streamflow regimes^7^,^8^. Populations in arid regions are heavily dependent on snow and glacier melt for their water supplies, with more than one-sixth of Earth’s population relying on glaciers and seasonal snowpacks^9^.

Global warming accelerates the water cycle, which has benefits for water availability^10^. However, in regions where water resources are dominated by glacier and snow melt from mountains, the availability is more complicated. Water supplies in these areas are strongly related to changes in temperature and precipitation, as well as changes in the snow and ice distributed across the mountains. Increases in temperature have important consequences for the hydrological cycle, particularly in areas dominated by glacier and snow melt^9^.

The Tienshan Mountains, with its status as “water tower of Central Asia”, is situated in the Eurasia hinterland, far from any ocean. The Tienshan Mountains is about 2500 kilometers long and 250–350 kilometers wide. It consists of a series of mountains, basins, and valleys, and boasts one of the most developed glacier mountains in the world^11^. The Tienshan Mountains feed the majority of the area’s rivers through a combination of ice-snow meltwater in the high mountains, precipitation in the mid-mountain forests, and fissure water in the low mountains.

The characteristics of the form, supply and conversion of the region’s water resources are unique in the world^12^. Previous studies showed that nearly all regions have experienced a warming trend, and that the average global temperature has increased 0.7 °C over the past hundred years^13^. This warming trend is especially obvious in the Tienshan Mountains^14^, where the average annual warming rate over the past half-century has been 0.34 °C/10a^15^. This is significantly higher than both the average global warming rate and that of the Northern Hemisphere during the same period. Of special note is that the temperature experienced a sharp increase in 1997, and since then has been in a state of high variability^16^. Following the sharp increase, temperatures have been extremely variable^17^. Furthermore, the warming is expected to significantly affect melt characteristics^18^,^19^, leading to a continuation of accelerated glacier and snow shrinkage in response to climatic changes over the past 20 years^11^,^20^. This may result in the feedback effects of reduced glacier/snow surface albedo, which will then break the surface glacier energy and mass balance, and cause serious changes in the local water cycle.

The Tienshan Mountains cover a large portion of Central Asia (Fig. 1a), spanning regions of Uzbekistan, to Kyrgyzstan, south-eastern Kazakhstan, and the Xinjiang Uyghur Autonomous Region in China^11^,^21^. Climate change accelerates the global water cycle, exacerbates extreme hydrological events, intensifies the uncertainties of water resources, and causes the water-based ecosystem in Central Asia to become increasingly fragile^16^. Changes in the spatial and temporal distribution of water resources due to climate change will cause mismatches between the resources and their distribution (i.e., the supply and demand factor), which will then become increasingly unbalanced and negatively affect productivity^17^. This in turn will impact the relationship between countries in Central Asia and the construction of the Silk Road economic belt.

The Tienshan Mountains span several countries and sub-regions, creating a decentralized political entity of complex multi-national and multi-ethnic forms. These characteristics hinder integrated research on the Tienshan Mountains, so information about the region in its entirety remains scarce. Some studies currently address the timing and evolution of expected glacier shrinkage, snow melt, and precipitation fraction changes^22^,^23^,^24^, but a comprehensive assessment of these interactional processes under the context of climate change is still lack. Therefore, based on existing data, this project presents a comprehensive perspective by addressing the following key questions: 1) What are the sub-regional differences and changes in water supplies in the Tienshan Mountains, Central Asia? 2) How does climate change affect the spatio-temporal changes of precipitation form, snow fraction, and glacier/snow melt water processes? 3) What are possible changes and uncertainties of water storage based on future climate scenarios?

## Results

### A conspicuous warming and fluctuant precipitation change

**Figure 1:**
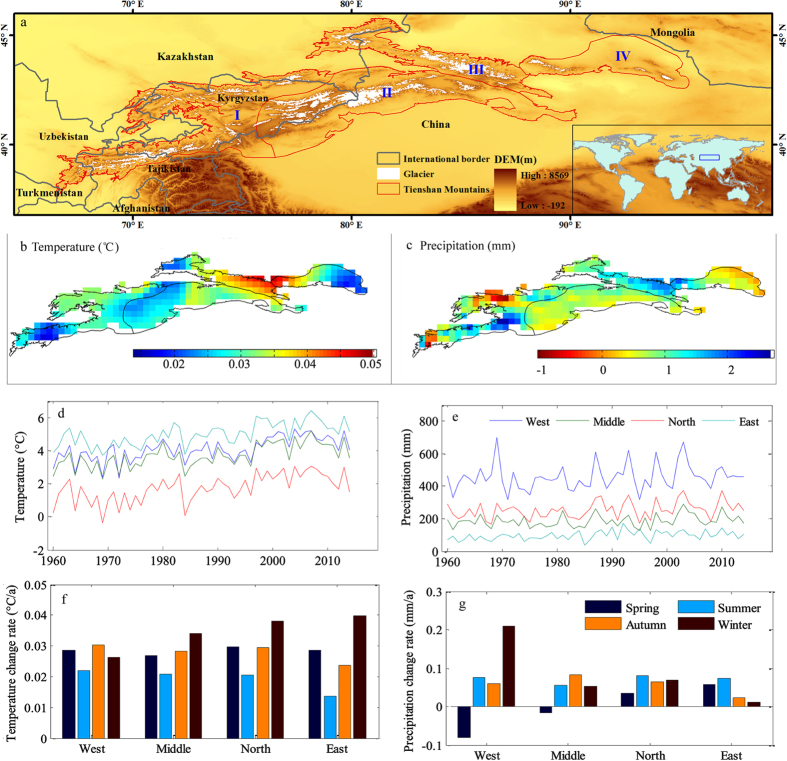
Climatic change in the Tienshan Mountains from 1960 to 2014. (**a**) The Tienshan Mountains are divided into four sub-regions: I. West Tienshan Mountains, II. Middle Tienshan Mountains, III. North Tienshan Mountains, and IV. East Tienshan Mountains^19^; (**b,c**) are the spatial change rates of temperature and precipitation; (**d,e**) are their inter-annual variation for different sub-regions; and (**f,g**) are the seasonal changes of temperature and precipitation. (Generated by ArcGIS 10.2, URL: http://www.esri.com/software/arcgis/arcgis -for-desktop).

Obvious warming in the Tienshan Mountains was detected at a rate of 0.3 °C/10a. Spatially, temperatures rose fastest in Middle and East Tienshan Mountains, at a rate of 0.45 °C/10a, while only slightly increasing in West Tienshan Mountains (Fig. 1b,d). Seasonally, temperatures increased more quickly in winter and spring than in summer (Fig. 1f).

Average annual precipitation in the Tienshan Mountains is about 290 mm, with significantly less precipitation in East Tienshan Mountains (97 mm) and much more in West Tienshan Mountains (455 mm). Although annual precipitation increased only slightly during the past half-century (Fig. 1c), there were clear increasing trends (1.2 mm/a) in the western and northern parts of the Tienshan Mountains, but only slight increasing trends (0.66 mm/a) in Middle Tienshan Mountains (Fig. 1e). Seasonal precipitation either increased insignificantly or decreased in spring, while demonstrating considerable increases in winter. Winter increases in precipitation were especially notable in West Tienshan Mountains, rising 23% over the past 55 years (Fig. 1g).

Increased temperatures in winter and spring are generally not beneficial to glacier or snow accumulation. The warmth leads to earlier snowmelt in mountain regions (especially in spring), thus leaving glaciers exposed to the air and radiation energy due to the decreased reflection ratio. As a consequence, glaciers are becoming easier to melt and more sensitive to warming^5^,^25^,^26^. Temperature increases are expected to affect precipitation and snowpacks in the mid to late part of the snow season^27^. One anticipated change is the temperature-induced shift of precipitation from snow to rain and the subsequent earlier melting of snowpacks^28^. Rising temperatures will cause a later start date for snow and an earlier end date^9^.

A shift in precipitation from snow to rain causes a decrease in snowfall fraction^24^. In Middle and East Tienshan Mountains, the snowfall fraction has decreased every decade, from 27% in 1960–1969 (first decade under study) to 25% in 2005–2014 (last decade under study). This is consistent with a study conducted by Guo and Li^29^, who used station meteorological data to conclude that the snowfall fraction in Middle Tienshan Mountains has declined. The decrease of snowfall fraction in the eastern part of the Tienshan Mountains may be caused by a relatively low precipitation increase rate coupled with a high temperature increase trend. The decline in the snowfall fraction could result in the reduction of snow and glacier accumulation in winter.

### Changes in snow cover

**Figure 2:**
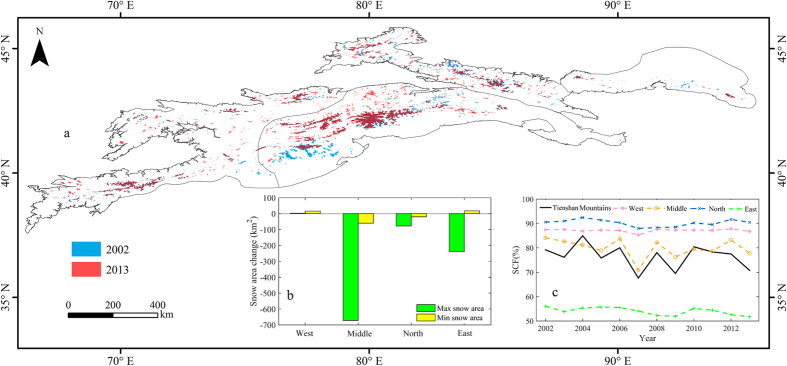
Snow cover changes in the Tienshan Mountains from 2002 to 2013. (**a**) Spatial distribution of snow cover in 2002 and 2013. The blue line is snow cover in 2002 and the red line is snow cover in 2013; (**b**) changes in maximum snow cover area and minimum snow cover area in sub-regions; (**c**) trend of maximum snow cover fraction (Max SCF) in 2002–2013. (Generated by ArcGIS 10.2, URL: http://www.esri.com/software/arcgis/arcgis-for-desktop).

From a climate perspective, changes in snowfall potentially translate into changes in snow cover^30^, and changes in mountain snow cover have a direct impact on water resources in arid regions^9^. Based on the MODIS data of snow cover in 2002–2013, we analyzed the snow cover area and maximum snow cover fraction in the Tienshan Mountains. The maximum and minimum snow cover areas represent the annual maximum and minimum snow cover areas, respectively. The results indicated that the snow cover area showed a decreasing trend in most parts of the Tienshan Mountains during 2002–2013 (Fig. 2a). Spatially, the snow cover areas in Middle Tienshan Mountains decreased significantly, with a decreased rate of −672 km^2^/a and −60 km^2^/a of the maximum and minimum snow cover areas, respectively. The loss areas accounted for 4.1% and 6.3% of the total loss areas in nearly 10 years. The loss rates of the maximum (−78 km^2^/a) and minimum (−20 km^2^/a) snow cover areas in North Tienshan Mountains were lower than those in Middle Tienshan Mountains. Meanwhile, the snow areas showed an increasing trend in West Tienshan Mountains, where the maximum and minimum snow cover areas increased at rates of 2.3 km^2^/a and 16 km^2^/a, respectively, or 0.01% and 4.8% (Fig. 2b).

The maximum snow cover fraction (SCF) is defined as the ratio of the area of maximum snow cover and the study area. North Tienshan Mountains has the highest maximum SCF (90%), followed by West Tienshan Mountains (87%), Middle Tienshan Mountains (79%), and East Tienshan Mountains (54%) (Fig. 2c). The maximum SCF decreased at a rate of −0.17%/a over the past 10 years. Spatially, Middle and East Tienshan Mountains had the largest decreases, showing rates of −0.32%/a and −0.28%/a respectively, followed by North Tienshan Mountains (−0.09%/a). Similar to changes in maximum snow cover area, the maximum SCF also increased only slightly in West Tienshan Mountains (0.01%/a).

### Substantial glacier shrinkage

**Figure 3:**
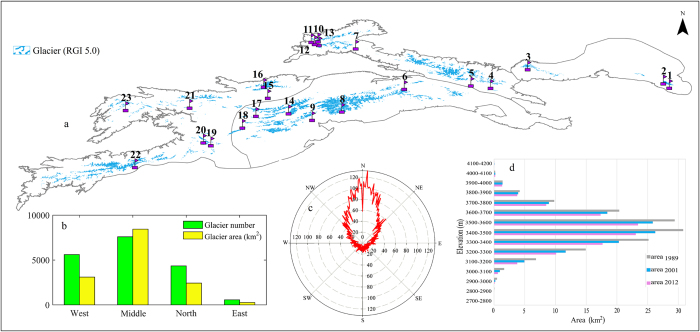
Changes and distribution of typical glaciers in catchments in the Tienshan Mountains in Central Asia. (**a**) Glaciers numbers of 1–23 based on the following studies: 1. Miaoergou: Xie *et al*.^53^, 2. East: Wang *et al*.^54^, 3. Bogda: Li *et al*.^32^, 4. Urumqi No. 1: WGMS REF Glaciers^55^, 5. East-central: He *et al*.^56^, 6. West-central: He *et al*.^56^, 7. North: He *et al*.^56^, 8. West: He *et al*.^56^, 9. Tomur: Huai *et al*.^57^, 10. Terisakkan: Kaldybayev *et al*.^26^, 11. Koksu: Kaldybayev *et al*.^26^, 12. Chizhin: Kaldybayev *et al*.^26^, 13. Kora: Kaldybayev *et al*.^26^, 14. Akshiirak: Kriegel *et al*. (2013)^58^, 15. Ili-Kungoy: Narama *et al*.^25^, 16. TS. Tuyuksuyskiy: WGMS REF Glaciers^55^, 17. Dzhetim: Kriegel *et al*.^41^, 18. At-Bashy: Narama *et al*.^25^, 19. SE-Fergana: Narama *et al*.^25^, 20. At-Bashi Kirkasi: Kriegel *et al*. 41, 21. Lower Nargn: Kriegel *et al*.^41^, 22. Abramov: Barandun *et al*.^58^, 23. Pskem: Narama *et al*.^25^; (**b**) glacier number and area in sub-regions (West, Middle, North, and East Tienshan Mountains); (**b**) glacier number and area in sub-regions (West, Middle, North, and East Tienshan Mountains); (**c**) distribution of glacier aspect; (**d**) distribution of glacier areas and their changes vs. elevation interval in the Karatal River Basin located in North Tienshan Mountains (ID = 10, 11, 12, 13). (Generated by ArcGIS 10.2, URL: http://www.esri.com/software/arcgis/ arcgis-for-desktop).

Glaciers are an important part of Central Asia’s water tower status. According to the latest statistics in the Randolph Glacier Inventory (RGI 5.0) (http://www.glims.org/RGI/rgi50_dl.html)^31^, there are about 10,778 glaciers in the Tienshan Mountains, covering a total area of about 13,566.6 km^2^. Glaciers in Middle Tienshan Mountains account for 59.3% of the total area (Fig. 3b). Most of the glaciers were north-facing (north, northwest and northeast) (Fig. 3c).

**Figure 4:**
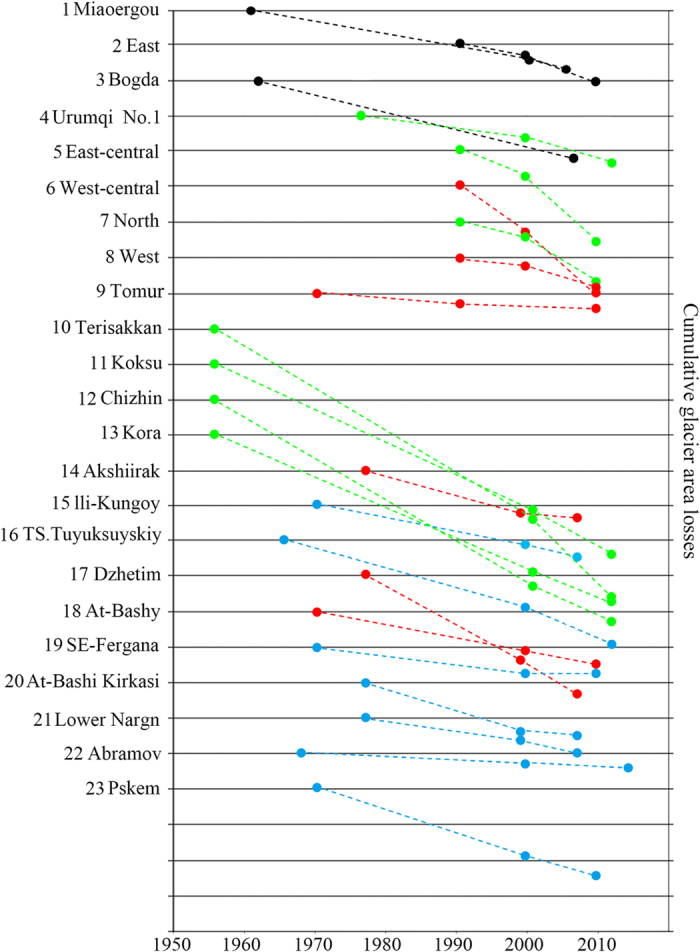
Recent shrinkage of selected glaciers in Tienshan Mountains. The colors represent sub-regions studies, with blue colors representing the West Tienshan Mountains, red colors representing the Middle Tienshan Mountains, green colors representing the North Tienshan Mountains, and black colors representing the East Tienshan Mountains. Lines represent 10% units, the first measurement equals 100% of glacier in the reference year. (Generated by Matlab 2012a, URL: http://cn.mathworks.com/products/matlab/).

Global warming has accelerated glacial melt in the Tienshan Mountains^32^,^33^. The results indicate that, over the past half-century, nearly all of the glaciers showed decreasing trends. Specifically, based on statistics of glacier changes in the 40 watersheds in the Tienshan Mountains^19^, we concluded that from 1960s to 2010s, about 97.52% of the glaciers retreated, while only 2.14% increased and 0.34% showed no obvious change. The results from our detailed analysis of glacier changes in 1960s–2010 show that, in 1960s–2000 time series, West Tienshan Mountains had the largest glacier retreat rate (−20%), followed by Middle Tienshan Mountains (−15.01%). Similarly, the glacier retreat rates in North Tienshan Mountains and Bogda Peak in the eastern part of the Tienshan Mountains were −13% and −3.1%, respectively (Fig. 4). Higher glacier degradation rates were reported for more recent decade compared to the mid 20th century for the North Tienshan Mountains and Bogda Peak in the eastern part of the Tienshan Mountains exhibited accelerated glacier retreat rates of −13.8% and −7.45%, respectively. The glacier retreat rates in West Tienshan Mountains and in the western part of Middle Tienshan Mountains are likely remained stable or even slightly decreased since 2000, with retreat rates of −8.1% and −10.1%, respectively.

Analysis of glacial shrinkage indicates a greater loss in smaller glaciers (<1 km^2^)^26^. About 98% of the glaciers in the Tienshan Mountains are small glaciers, which are more sensitive to climate change than large glaciers^26^. Hence, glacier loss was significant, especially for smaller glaciers in the Bogda Peak, in the eastern part of the Tienshan Mountains. However, it is noteworthy that absolute glacier mass balance loss was higher for larger glaciers. Overall, the melting of glaciers has had a significant impact on water resources.

The analysis results of glacier changes at different altitudes indicate that all elevations showed a reduction in glacier mass balance. Retreat rates for glaciers located at lower altitude regions were faster than retreat rates at higher altitudes. Figure 3d illustrates the changes in 1989, 2001 and 2012 in 243 glaciers in the Karatal River Basin, located in North Tienshan Mountains. The results show that below the elevation of 3600 m, the glacier retreat rate is about −27%, while above that elevation the retreat rate is −16%. The number of glaciers in the Karatal River Basin was reduced from 243 in 1989 to 214 in 2012, while the glacier area decreased from 142.8 km^2^ to 109.3 km^2^, or about −23.45%^26^.

### Runoff changes in the Tienshan Mountains

**Figure 5:**
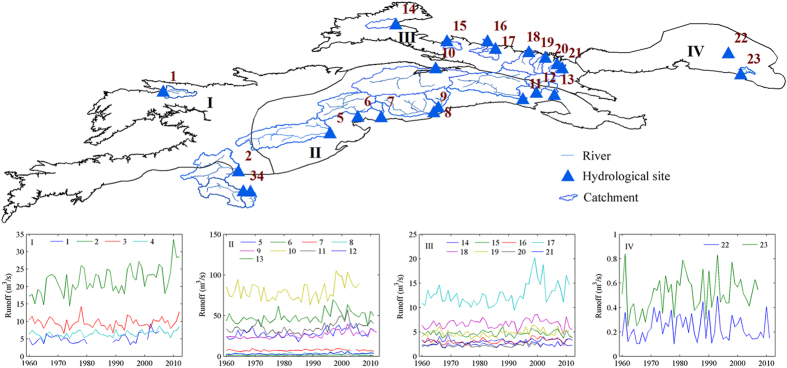
Runoff variations for typical rivers in the (I) West Tienshan Mountains, (II) Middle Tienshan Mountains, (III) North Tienshan Mountains, and (IV) East Tienshan Mountains. The location of hydrological stations: 1, Karaoy; 2, Karabaly; 3, Kelak; 4, Shaman; 5, Shaliguilank; 6, Xiehela; 7, Tailan; 8, Heizi; 9, Heizi Reservoir; 10, Kafuqihai; 11, Dashankou; 12, Huangshuigou; 13, Kerguty; 14, Wenquan; 15, Jingheshankou; 16, Jilede; 17, Jiangjunmiao; 18, Kenwast; 19, Shimen; 20, Zhicaichang; 21, Yingxiongqiao; 22, Erdaogou; 23, Toudaogou. (Generated by ArcGIS 10.2, URL: http://www.esri.com/software/arcgis/arcgis- for-desktop, and Matlab 2012a, URL: http://cn.mathworks.com/products/matlab/).

Under the context of global warming, the vulnerability of water systems and uncertainty of water resources are increasing^17^. Runoff variations of typical rivers in the Tienshan Mountains since 1960 were shown in Fig. 5. Except the East Tienshan Mountains (IV), the runoffs of typical rivers in the other three sub-regions showed an increasing trend since 1960, especially obvious in the Middle Tienshan Mountains, where runoffs increased significantly (*p* = 0.01) for most stations.

River runoff responds in a complex way to changes in climate and in the cryosphere. Snow and glacier melt are believed to substantially contribute to spring and summer runoff providing water. The studies on runoff trends in the Tienshan Mountains indicated a complex response of catchments to climate changes. It appears that catchments with a higher fraction of glacierized area showed mainly increasing runoff trends in the past (e.g., Middle Tienshan Mountains), while river basins with less or no glacierization exhibited large variations in the observed runoff changes (e.g., East Tienshan Mountains). The cryosphere is widely acknowledged to be an important water storage component in Central Asia contributing substantially to river runoff. Variations in the observed runoff increased apparently for most rivers, which will likely make the hydrological processes much more complexity triggered by climatic and cryospheric changes, more uncertainties are expected in runoff prediction in the glacier/snow melt and precipitation recharged rivers.

### Terrestrial total water storage variations

**Figure 6:**
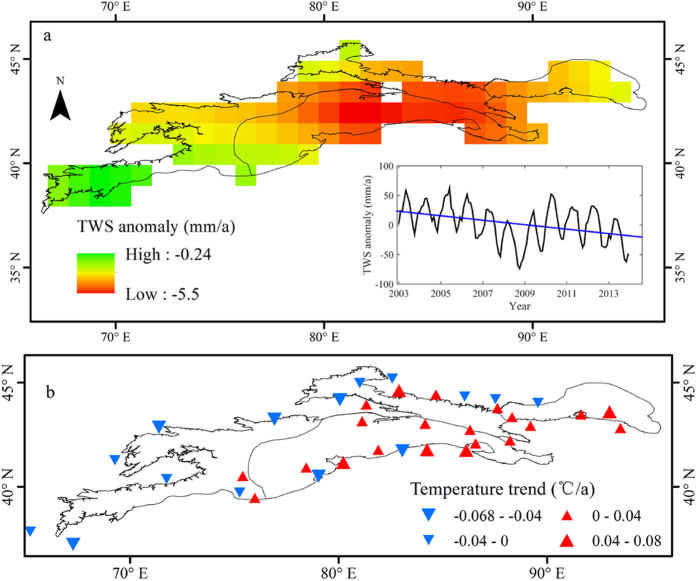
(**a**) Terrestrial total water storage changes in the Tienshan Mountains in 2003–2013; (**b**) trend of annual temperature of meteorological sites in the Tienshan Mountains in 2003–2013. (Generated by ArcGIS 10.2, URL: http://www.esri.com/software/arcgis/arcgis-for-desktop).

Glaciers and seasonal snow cover are expected to change their water storage capacity under the ongoing warming of the global climate. This will have major consequences for downriver water supplies^34^,^35^. Based on GRACE data of terrestrial total water storage (TWS) variations in the Tienshan Mountains in 2003–2013, the results indicate a decreasing trend in TWS, with a decline rate of −3.72 mm/a (Fig. 6). This suggests that the water tower loss in Central Asia has been about −2.23 × 10^8^ m^3^/a over the past 10 years.

A decreasing trend in TWS was also observed in Middle Tienshan Mountains over the past 10 years, falling at a rate of −5.5 mm/a (Fig. 6). Furthermore, both the winter and summer halves of the year exhibited a significant decreasing trend at a rate of −5.72 and −5.92 mm/a, respectively. Meanwhile, TWS variations revealed only a slight decreasing trend in West Tienshan Mountains, at a rate of −0.12 mm/a, which includes an increasing trend of 0.03 mm/a in the winter half of the year.

In the Tienshan Mountains, changes in total water storage are closely related to changes in glacier/snow distribution and climatic factors such as temperature and precipitation. Figure 6a shows that TWS in Middle Tienshan Mountains underwent a significant decreasing trend over the past decade, with the largest decrease measuring −5.5 mm/a. This sub-region also has the most developed glaciers. The number, area, and volume of glaciers in Middle Tienshan Mountains account for 41.9%, 59.3% and 74.9%, respectively, of all the glaciers in the Tienshan Mountains.

In West Tienshan Mountains, however, the decrease in TWS was slower, and the winter half of the year even shows an increasing trend rate of 0.03 mm/a. These variations are closely related to temperature and precipitation changes in the sub-regions. Figure 6b illustrates that temperature has a significant increasing trend east of the 80°E region, rising at a rate of 0.4–0.8 °C/10a, whereas in the western part of the Tienshan Mountains, the temperature rise is not so obvious. Some areas even show a declining trend.

Overall, the data indicates that the rapid warming in the Tienshan Mountains over the past half-century has accelerated glacier shrinkage and snow melt, changed the fraction of rainfall and snowfall, reduced the snowfall fraction and glacier accumulation, and led to a significant reduction in total water storage.

### Future changes in terrestrial total water storage

**Figure 7:**
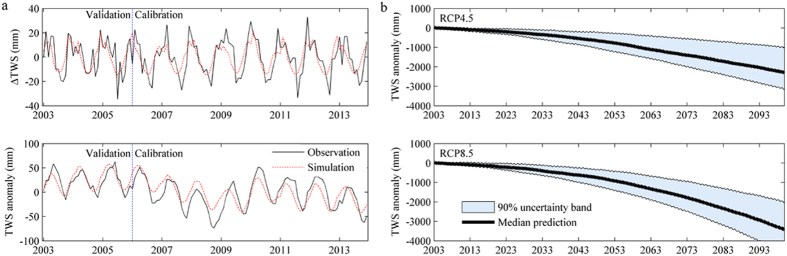
(**a**) Observed and simulated ∆TWS and TWS anomaly for calibration and validation periods in the Tienshan Mountains; (**b**) predicted future water storage changes in the Tienshan Mountains using a 21-GCM ensemble in CMIP5 under RCP4.5 and RCP8.5. The 90% uncertainty bands are shown in shaded areas. (Generated by Matlab 2012a, URL: http://cn.mathworks.com/products/matlab/).

A 21-GCM ensemble from CMIP5 was used to predict future water storage in the Tienshan Mountains. The results show that the simulated ΔTWS_i_ agreed well with the GRACE-detected ΔTWS_i_, with NS and R^2^ being 0.57 and 0.59 for the calibration period (2006–2013) and 0.58 and 0.62 for the validation period (2003–2005). This indicates that the meteorological inputs (i.e., temperature and snowfall) could account for about 60% of gravity change. The model can be used to roughly analyze future variations in water storage in the Tienshan Mountains (Fig. 7a).

The median prediction indicated that water storage will slight decrease until the 2040s, but will then experience a large deficit in the latter half of the 21^st^ century, especially under RCP8.5 (Fig. 7b). The average decreasing trend rates for water storage are −0.25 (the 10% and 90% quintiles are −0.33 to 0.11) and −0.35 (the 10% and 90% quintiles are −0.46 to −0.22) m/yr, which is similar to the current water loss rate. It is also similar to the adjacent Hindu Kush area (−0.12 ± 0.16 m/yr)^36^, but higher than 0.06 ± 0.005 m/yr^37^. The 90% uncertainty bands widen as the prediction period lengthens. For example, TWS ranges from −3500 mm to −1000 mm under RCP4.5, indicating significant uncertainty not only in magnitude but in overall changing direction compared to the current period. Note that TWS demonstrates a constant decreasing trend from the 2040s onwards for all GCM models under both scenarios, which may pose great danger for the water tower and influence the water supply for the oasis and desert regions.

This study approximated changes in water storage in the Tienshan Mountains using the degree-day method and gravity anomaly. There were some uncertainties in the prediction due to the assumption that meteorological variables account for all of the GRACE signals, although Arendt *et al*.^38^ indicated that meteorological variation-induced glacier change contribute more than 50% of GRACE signals. In addition, significant uncertainty levels can be found in the GCM ensemble, which are inferred in the wide uncertainty range in Fig. 7.

## Discussion

The Tienshan Mountains are the water tower of Central Asia as well as the region’s main water source and ecological barrier^19^. Recent observational air temperature analyses showed that temperatures in and around the Tienshan Mountains experienced a sharp increase over the past half-century. Rapid warming affects the energy and mass balance of glacier surfaces, alters the original glacier/snowmelt water processes, accelerates the melting of glaciers, snow and permafrost, and causes the mutual feedback of decreased surface albedo of glacier/snow and glacier/snowmelt water, thereby affecting the recharge of runoff and water resources.

Studies on global climate change and the water cycle indicate that total water storage plays a major role in these processes. Our results showed that TWS experienced a decreasing trend across the entire Tienshan Mountains region, which is consistent with Yang’s study of changes in TWS in the Chinese Tienshan Mountains^39^. TWS also experienced a significant decreasing trend in Middle and East Tienshan Mountains, but only a slight decreasing trend in West Tienshan Mountains. Furthermore, TWS changes in the Tienshan Mountains are closely related to changes in glacier/snow distribution and climatic factors such as temperature and precipitation. Glaciers and seasonal snow cover are expected to change their water storage capacity under the ongoing warming of the global climate, with major consequences for downriver water supplies^34^,^35^. Spatially, the regions of significantly decreased TWS are similar to the regions of accelerated glacier shrinkage and decreased snow cover.

The glacier mass balance changes in the investigated glaciers confirmed an expected and widely published trend of glacier shrinkage. Where multiple surveys are available, most reveal an accelerating loss^11^,^40^. The results of previous studies show large variations in different parts of the Tienshan Mountains: −0.76% a^−1^ from the mid-1970s to the mid-2000s in West and North Tienshan Mountains^22^,^41^; −0.11% a^−1^ in 1975–2008 in Middle Tienshan Mountains^42^; and −0.35% a^−1^ in 1963–2000 in East Tienshan Mountains^43^. However, despite a shrinkage of −20% in 1960–2000 in the western parts of the Tienshan Mountains, our results showed a comparatively lower shrinkage rate of −8.1% in 2000–2012 in here. Changes in temperature and precipitation in the Tienshan Mountains sub-regions are the dominant factors contributing to glacier mass balance variation^44^. Ice mass balance is controlled by precipitation type (liquid or solid) and surface albedo^5^, both of which are sensitive to temperature. In High Asia, summer freezing level height (FLH) has shown a predominantly upward trend in 1958–2005, consistent with the retreat of the cryosphere^45^. Moreover, regions with mostly small glaciers are generally more sensitive to climate change because smaller glaciers have a shorter response time to climate change^46^. Since melt and accumulation show their highest rates during the same season, temperature controls both processes. This highlights the vulnerability of glaciers in the Tienshan Mountains with respect to temperature changes. At this pace, half of the total glacier ice volume estimated to be present in the Tienshan Mountains today will be lost by the 2050s^19^.

Consistent with decreases in glaciers, the snow cover in the Tienshan Mountains has likewise decreased. The maximum and minimum snow cover areas and maximum snow cover fraction are closely related to changes in temperature and precipitation^9^. In Middle and East Tienshan Mountains, the maximum snow cover fraction has decreased significantly. However, in West Tienshan Mountains, the maximum snow cover fraction has increased, albeit slightly. This phenomenon is related to the warming hiatus that has occurred in the western part of the Tienshan Mountains, whereas the eastern part has experienced rapid warming. In regions where the land surface hydrology is dominated by winter glacier/snow accumulation and spring melt, the performance of water management systems is more strongly related to temperature than to precipitation changes^9^.

One anticipated change is a temperature-induced shift of precipitation from snow to rain and an earlier melt of the snowpacks^28^. This will cause a delay in the start of the snow season and an early end to it^9^. The expected decrease in snowfall fraction in the eastern part of the Tienshan Mountains may be caused by a relatively high temperature increasing trend (0.45 °C/10a), while in the western part of the Tienshan Mountains, the snowfall fraction may experience only a slight increasing trend due to the warming hiatus. Changes in snowfall fraction also affect the accumulation and melting processes of glaciers^8^, which thus affects the total water storage and water resources. Our results show that, over the past decade, Middle and East Tienshan Mountains experienced decreased snowfall fractions in the areas with the fastest glacial recession (e.g., Nos 5–8 in Fig. 3). In contrast, glaciers Nos 14, 17, 19 and 20 in Fig. 3 had a slower rate of glacier shrinkage, and snowfall fractions in these areas showed either no obvious changes or only slight increases.

The snow fraction has a significant effect on water resources. An increased fraction of precipitation as snowfall is associated with higher streamflow^24^. However, little work has yet been done to investigate the impact and mechanism of this shift of precipitation on runoff, which is a key factor that controls the available freshwater resources for domestic and agricultural needs. In situations of slight precipitation change, the snowfall fraction restraint of the runoff processes is unknown, as is the mechanism that causes it. This information is important for the estimation of changes in future water resources under various scenarios. The predicted ongoing warming and further reduction in snowfall fraction^30^ will inevitably influence the accumulation and melting processes of snow and glaciers, which will then further influence the streamflow and terrestrial total water storage in the Tienshan Mountains^24^. If glaciers and snow continue to decrease, water storage will likewise decrease. This may lead, within just a few decades, to some rivers running out of water in the dry season^9^.

## Methods

### Test for each observation station

A significance test was took place in the following steps: 1) eliminating source data for stations whose location is unknown or questionable (they identified with a location name, latitude, and longitude using the metadata associated with the source dataset or from other standard station history information); 2) stations with missing observations for more than 5 consecutive days or with 20% observation missing in any 30-day period were excluded; 3) all data should pass the standard normal consistency check.

### Snowfall fraction algorithm

Precipitation for a certain month is considered to be entirely snowfall if the corresponding temperature is less than 1 °C. Otherwise, it is rainfall^24^.

### Maximum snow cover fraction (SCF)

We used the maximum snow cover area, minimum snow cover area, and maximum snow cover fraction (SCF) to analyze snow cover changes in the Tienshan Mountains in 2002–2013.


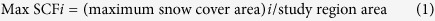


where *i* = 2002, 2003, …, 2013; Max SCF*i* represents the maximum snow cover fraction of *i* year.

### Total water storage algorithm

The equivalent water height is calculated by the following equation^47^:


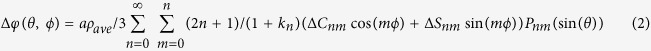


where *φ* is the equivalent water height, *θ* is the latitude, *ϕ* is the longitude, a is the equatorial radius, *ρ*_*ave*_ is the mean density of Earth, *k*_*n*_ is the love number, *C*_*nm*_ and *S*_*nm*_ are the coefficients of the spherical harmonics (Stokes’ coefficients), and *P*_*nm*_ (sin(*θ*)) is the *n*_*th*_ degree and *m*_*th*_ order of the fully-normalized Legendre function.

The first step was to calculate the mean monthly gravity field spherical harmonics coefficients for all 138 months of GRACE solutions from Apr 2002 to Oct 2014. The second step was to compute the time-variable gravity field spherical harmonics coefficients, which uses each monthly value to subtract the mean monthly gravity field spherical harmonics coefficients. Next, the decorrelation algorithm supported by Duan *et al*.^48^ was used to remove the longitudinal stripe error effects. As a final step, we applied the GRACE filter using a Gaussian averaging filter^48^ with a smoothing radius of 300 km to calculate the changes in TWS in the Tienshan Mountains in Central Asia based on changes in Earth’s gravity field.

It is difficult, at present, to use ground truth to validate GRACE mass estimates, but it is able to effectively reveal the trend of total water storage in lager regions^49^, i.e. the Tienshan Mountains. In this study, the estimated measurement error is about 30 mm in GRACE TWS in the entire Tienshan Mountains. Yang and Chen^39^ take consideration of the leakage errors to calculate the uncertainty of TWS is about 4.3 ± 1.2 mm/yr in the TienShan Mountains in China, which looks consistent with us.

### Prediction of total water storage

The climate change projections from 21 state-of-the-art GCMs from CMIP5 were used to investigate climatic changes in the Tienshan Mountains. However, the use of multiple GCMs and RCPs can inject uncertainties into climatic projections. The GCMs used include BCC-CSM1.1-m, CanESM2, CMCC-CM, CNRM-CM5ACCESS1.3, CSIRO-Mk3.6.0, BNU-ESM, INM-CM4, IPSL-CM5B-LR, FGOALS-g2, MIROC5, MIROC-ESM, HadGEM2-ES, MPI-ESM-LR, MRI-ESM1, GISS-E2-R, CCSM4, NorESM1-M, GFDL-CM3, GFDL-ESM2G, and CESM1(BGC). The projected future climate change (including air temperature and snowfall) was analyzed under two emission scenarios of the Representative Concentration Pathways (RCP) of RCP4.5 (lower emission scenario) and RCP8.5 (higher emission scenario). The RCP4.5 is a stabilization scenario with the total radiative forcing rising until 2070, and then remaining at a stable centration of 4.5 W/m^2^. In contrast, RCP8.5 is a continuously rising radiative forcing pathway (at a target of 8.5 W/m^2^ in 2100) with a further enhanced residual circulation and significant CO_2_ and CH_4_ increases^50^.

To gauge possible future changes in terrestrial total water storage, we built a model by calibrating three parameters (*β*_*0*_*, d, T*_*mlt*_) against the mass balance determined from GRACE data. The Nash-Sutcliffe coefficient^51^ was used as the objective function. The gravity anomaly signal from the GRACE data set is suitable for assessing changes in glacier mass budget (e.g. refs 36 and 38), especially for regions with high proportions of glaciers and permanent snow. Therefore, we assume the water storage change is mainly attributed to the accumulation and melting of glaciers and snow. In this study, gravity changes caused by variations in groundwater, soil water, and the uplift of the Tienshan Mountains were not considered. The simple but practical degree-day model, which generally provides robust estimates of glacier and snow mass changes^52^, is used to calculate the variation of water storage in the Tienshan Mountains.


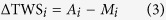



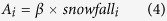



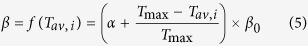



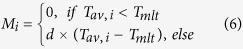


where ΔTWS_i_ is the change in glacier and snow mass for the i^th^ month compared to the previous month (in this case, ΔTWS_i_ = *A*_*i*_*−M*_*i*_ is calculated as the difference of GRACE-interpreted TWS anomalies for the i^th^ month and the previous month), and *A*_*i*_ and *M*_*i*_ represent the accumulation and melt of glacier and snow. *A*_*i*_ is calculated using *snowfall*_*i*_ and conversion coefficient β, while β is related to temperature (T_max_ refers to the maximum temperature). Higher temperatures result in low β, and less snowfall converting to glacier and snow cover. α is a coefficient used to reduce the impact of temperature on glacier accumulation, here we assume α = 0.4, with d being the melt factor and T_mlt_ the melt base temperature.

### Data sources

Due to the scarcity of meteorological sites data in the Tienshan Mountains, the CRU TS3.22 dataset (http://www.cru.uea.ac.uk/cru/data/hrg/) was used to detect changes in precipitation and temperature since 1960.

The snow cover data was obtained from the MODIS/Terra Snow Cover 8-Day L3 product (MOD10A2, version 5). These data are produced every eight days at gridded resolutions of 500 meters and are supported by the NASA National Snow and Ice Data Center Distributed Active Archive Center. A total of 4,575 images were used from Jan 2002 to Feb 2013 (http://dx.doi.org/10.5067/C574UGKQQU1T).

The glacier information was obtained from the World Glacier Monitoring Service (WGMS, available at http://nsidc.org/data/glacier_inventory/) and China Glacier Inventory (CGI) from the West Data Central (available at http://westdc.westgis.ac.cn/). The glacier information is also supported by the Randolph Glacier Inventory (RGI 5.0). The RGI is an inventory of global glacier outlines that serves as a supplement to the Global Land Ice Measurements from the Space initiative (GLMS is available at http://www.glims.org/RGI/index.html). The glacial mass balance information is from Farinotti’s^19^ research results.

The Gravity Recover and Climate Experiment (GRACE) Mission is a collection of low Earth orbit satellites, jointly developed by the United States and Germany, and launched in March, 2002. The GRACE Mission has carried out more than ten years of continuous observation of global gravitational field changes. The observation data has been widely used to study changes in terrestrial water, the Antarctic and Greenland ice sheets, and global sea levels. After years of development, the theory and methods of changes in terrestrial water storage are relatively mature by GRACE data^49^.

## Additional Information

**How to cite this article**: Chen, Y. *et al*. Changes in Central Asia’s Water Tower: Past, Present and Future. *Sci. Rep.* **6**, 35458; doi: 10.1038/srep35458 (2016).
