# Corrigendum: Changes in Central Asia’s Water Tower: Past, Present and Future

**DOI:** 10.1038/srep39364

**Published:** 2016-12-22

**Authors:** Yaning Chen, Weihong Li, Haijun Deng, Gonghuan Fang, Zhi Li

Scientific Reports 6: Article number: 3545810.1038/srep35458; published online: 10 20 2016; updated: 12 22 2016

This Article contains a typographical error in the Results section under subheading ‘Terrestrial total water storage variations’.

“This suggests that the water tower loss in Central Asia has been about −2.23 × 10^8^ m^3^/a over the past 10 years.”

should read:

“This suggests that the water tower loss in Central Asia has been about −2.23 × 10^9^ m^3^/a over the past 10 years.”

